# Supplementary material for: Sustainability of coral reefs are affected by ecological light pollution in the Gulf of Aqaba/Eilat
Source: Commun Biol. 2019 Aug 5;2:289. doi: 10.1038/s42003-019-0548-6 (PMC6683144; doi:10.1038/s42003-019-0548-6)
Supplement: Supplementary file 1 — Supplementary Information [file 42003_2019_548_MOESM1_ESM.pdf]

**Supplementary figures**

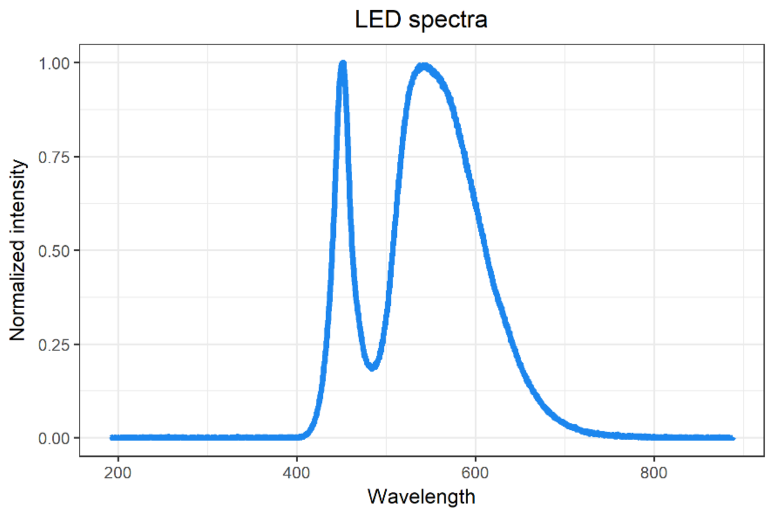


Supplementary Figure 1: **LED light spectrum** showing wave length and intensity made by using the Ocean Optics JAZ spectrometer. the curve is normalized to be 1 at its maximum.

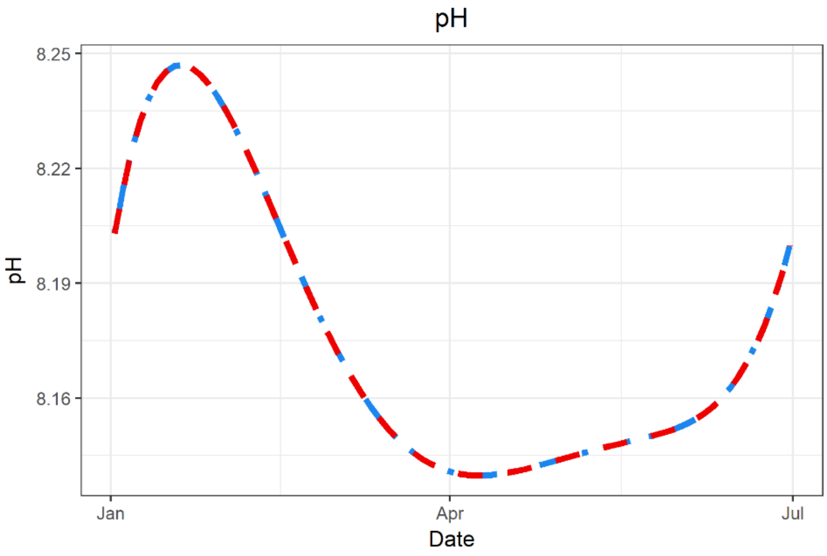


Supplementary Figure 2: **pH measurements** in both aquariums for the duration of the experiment (February-June). Red dotes indicate control (AMB) tank, blue dots indicate light treatment (ELP) tank.

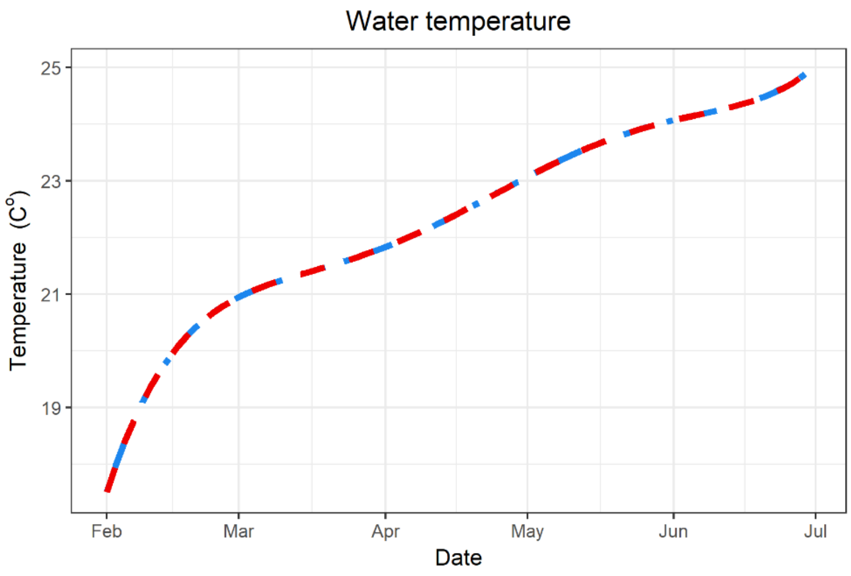


Supplementary Figure 3: **Temperature measurements** in both aquariums for the duration of the experiment (February-June). Red dotes indicate control (AMB) tank, blue dots indicate light treatment (ELP) tank.

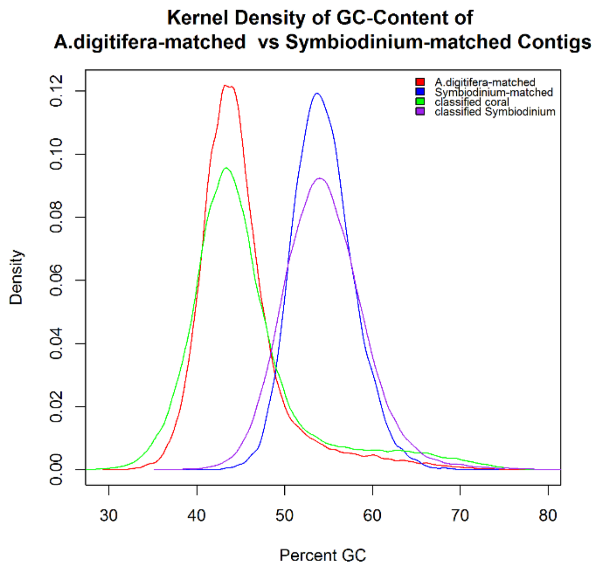


Supplementary Figure 4: **G-C content** of coral transcript and Symbiodinium transcripts showing the separation between the organism's transcripts.
