# Supplementary material for: Sustainability of coral reefs are affected by ecological light pollution in the Gulf of Aqaba/Eilat
Source: Commun Biol. 2019 Aug 5;2:289. doi: 10.1038/s42003-019-0548-6 (PMC6683144; doi:10.1038/s42003-019-0548-6)
Supplement: Supplementary file 3 — Reporting Summary [file 42003_2019_548_MOESM3_ESM.pdf]

nature research

Corresponding author(s): Yael Rosenberg and Oren Levy

Last updated by author(s): Jul 8, 2019

Reporting Summary

Nature Research wishes to improve the reproducibility of the work that we publish. This form provides structure for consistency and transparency in reporting. For further information on Nature Research policies, see Authors & Referees and the Editorial Policy Checklist.

Statistics

For all statistical analyses, confirm that the following items are present in the figure legend, table legend, main text, or Methods section.

n/a	Confirmed	
☐	☒	The exact sample size (*n*) for each experimental group/condition, given as a discrete number and unit of measurement
☐	☒	A statement on whether measurements were taken from distinct samples or whether the same sample was measured repeatedly
☐	☒	The statistical test(s) used AND whether they are one- or two-sided<br>*Only common tests should be described solely by name; describe more complex techniques in the Methods section.*
☐	☒	A description of all covariates tested
☒	☐	A description of any assumptions or corrections, such as tests of normality and adjustment for multiple comparisons
☐	☒	A full description of the statistical parameters including central tendency (e.g. means) or other basic estimates (e.g. regression coefficient) AND variation (e.g. standard deviation) or associated estimates of uncertainty (e.g. confidence intervals)
☐	☒	For null hypothesis testing, the test statistic (e.g. *F*, *t*, *r*) with confidence intervals, effect sizes, degrees of freedom and *P* value noted<br>*Give P values as exact values whenever suitable.*
☒	☐	For Bayesian analysis, information on the choice of priors and Markov chain Monte Carlo settings
☐	☒	For hierarchical and complex designs, identification of the appropriate level for tests and full reporting of outcomes
☐	☒	Estimates of effect sizes (e.g. Cohen's *d*, Pearson's *r*), indicating how they were calculated

Our web collection on statistics for biologists contains articles on many of the points above.

Software and code

Policy information about availability of computer code

Data collection	N/A
Data analysis	N/A

For manuscripts utilizing custom algorithms or software that are central to the research but not yet described in published literature, software must be made available to editors/reviewers. We strongly encourage code deposition in a community repository (e.g. GitHub). See the Nature Research guidelines for submitting code & software for further information.

Data

Policy information about availability of data

All manuscripts must include a data availability statement. This statement should provide the following information, where applicable:

- Accession codes, unique identifiers, or web links for publicly available datasets
- A list of figures that have associated raw data
- A description of any restrictions on data availability

The sequencing data reported in this study has been deposited to the Sequence Read Archive (SRA), under accession SRP135621. Correspondence and request for materials should be addressed to Y.R. yaelirose@gmail.com or O.L. oren.levy@biu.ac.il

Field-specific reporting

Please select the one below that is the best fit for your research. If you are not sure, read the appropriate sections before making your selection.

☐ Life sciences ☐ Behavioural & social sciences ☒ Ecological, evolutionary & environmental sciences

Ecological, evolutionary & environmental sciences study design

All studies must disclose on these points even when the disclosure is negative.

Study description	en mature colonies of Acropora eurystoma (measuring > 50 cm in diameter) were collected from 4-5-meter depth in the Gulf of Aqaba/Eilat Red Sea (28.6929° N, 34.7299° E) in February 2014. Corals were divided in two groups and placed in flow-through aquarium systems in the Inter-University Institute (IUI) in Eilat. The aquarium systems are part of the Red Sea Simulator (RSS), an outdoor system that is exposed to full spectrum ambient sunlight (1800 µmol m-2 s-1 PAR at midday on a cloudless day,) with a 15 h photo-period on the longest day and 11 h on the shortest day 21. Seawater temperature and pH (Polilyte Plus H Arc 120, Hamilton) were monitored and tested every hour through automated monitoring (sensor-carrying robot) to ensure all corals were under the same conditions (Table 1 and figures S2-3). Each experimental group, consisting of five coral colonies, was under a specific light regime; the first was natural light, ambient light cycle and moon phase (AMB corals). The second group had artificial light contamination (ELP corals) from small White LED light strips 6000-6500K (4.8 mol quanta m-2 s-1 at the top of the aquarium and 1.5-2 µmol quanta m-2 s-1 at coral height) that were placed behind the glass walls and were turned on every day at sunset.
Research sample	two groups (n=10)of the coral Acropora eurystoma
Sampling strategy	each sampling point all colonies were sampled
Data collection	coral fragments were collected by Yael Rosenberg and transfered to Bar-Ilan University in liquid nitrogen
Timing and spatial scale	sampling started at the full moon day in June 2014. Second sampling day was during the new moon day of June 2014. At each sampling day there were four sampling times: sunrise, noon, sunset and high moon. In total there were two sampling days, June 13th 2014 (full moon) and June 25th 2014 (new moon) and eight sampling times, four for each sampling day.
Data exclusions	no data was excluded
Reproducibility	all samples showed the same values of RNA extraction and RNA-Seq
Randomization	at each sampling time the same colonies were sampled. there was no randomization
Blinding	not relavent, same colonies were sampled

Did the study involve field work? ☒ Yes ☐ No

Field work, collection and transport

Field conditions	coral fragments were kept in flow-through aquarium systems in the Inter-University Institute (IUI) in Eilat. The aquarium systems are part of the Red Sea Simulator (RSS), an outdoor system that is exposed to full spectrum ambient sunlight
Location	study took place in the Inter-University Institute (IUI) in Eilat, Israel (28.6929° N, 34.7299° E)
Access and import/export	Corals were collected under the Israel Nature and National Parks Protection Authority permit number 2014/40227 issued to Yael Rosenberg and Oren Levy.
Disturbance	no disturbance

Reporting for specific materials, systems and methods

We require information from authors about some types of materials, experimental systems and methods used in many studies. Here, indicate whether each material, system or method listed is relevant to your study. If you are not sure if a list item applies to your research, read the appropriate section before selecting a response.

Materials & experimental systems

n/a	Involved in the study	
☒	☐	Antibodies
☒	☐	Eukaryotic cell lines
☒	☐	Palaeontology
☐	☒	Animals and other organisms
☒	☐	Human research participants
☒	☐	Clinical data

Methods

n/a	Involved in the study	
☒	☐	ChIP-seq
☒	☐	Flow cytometry
☒	☐	MRI-based neuroimaging

Animals and other organisms

Policy information about studies involving animals; ARRIVE guidelines recommended for reporting animal research

Laboratory animals	N/A
Wild animals	N/A
Field-collected samples	coral fragments were kept in flow-through aquarium systems in the Inter-University Institute (IUI) in Eilat. The aquarium systems are part of the Red Sea Simulator (RSS), an outdoor system that is exposed to full spectrum ambient sunlight
Ethics oversight	Corals were collected under the Israel Nature and National Parks Protection Authority permit number 2014/40227 issued to Yael Rosenberg and Oren Levy.

Note that full information on the approval of the study protocol must also be provided in the manuscript.
